# Association of epicardial adipose tissue thickness and left ventricular functions in children with primary dyslipidemia

**DOI:** 10.1007/s00431-026-06997-1

**Published:** 2026-05-07

**Authors:** Mohamed A. Hassan, Mina Mamdouh, Faisal-Alkhateeb Ahmed, Khaled Mohammed Allam

**Affiliations:** Faculty of Medicine, Assiut University, Assiut, Egypt

**Keywords:** Dyslipidemia, Epicardial Fat, Children, Left ventricular function

## Abstract

Epicardial adipose tissue (EAT) is a component of visceral adiposity and mediates cardiac function and atherosclerosis via expression of several bioactive molecules**.** To evaluate the significance and relationship between epicardial fat thickness (EFT) and familial dyslipidemia and left ventricular function. This prospective case–control study was conducted at Assiut University Children’s Hospital between September 2023 and August 2025. Twenty-one children with familial dyslipidemia and twenty-one age-, sex-, and BMI-matched healthy controls underwent clinical evaluation, lipid profile assessment, and transthoracic echocardiography, including measurement of epicardial fat thickness and left ventricular systolic and diastolic function according to American Society of Echocardiography guidelines. Dyslipidemic patients showed significantly higher total cholesterol (332.9 ± 222.3 mg/dL), triglycerides (391.4 ± 251.6 mg/dL), and LDL (154.3 ± 130.4 mg/dL) than controls (p < 0.001). Mixed hyperlipidemia was the most common type (47.6%). Echocardiography revealed increased epicardial fat thickness (2.88 ± 0.94 mm vs. 2.29 ± 0.57 mm; *p *= 0.018), larger left atrial (21.45 ± 3.86 mm; *p *= 0.031) and aortic diameters (17.54 ± 3.12 mm; *p *= 0.013). Triglyceride level was the only independent predictor of epicardial fat thickness (β = 0.437, *p *= 0.028).

*Conclusion*: Echocardiography revealed increased epicardial fat thickness and early cardiac remodeling. Serum triglycerides were the only independent predictor of EFT, suggesting its key role in subclinical cardiovascular risk among dyslipidemic children.

**Table:** 

**What is Known:** • *Epicardial adipose tissue is associated with cardiovascular risk factors in adults.* • *Children with primary dyslipidemia may develop early cardiac dysfunction.* **What is New:** • *This study demonstrates a signifi cant association between epicardial adipose tissue thickness and left ventricular function in children.* • *It highlights the potential role of epicardial fat as an early marker of cardiac involvement in pediatric dyslipidemia.*

**What is Known:**

• *Epicardial adipose tissue is associated with cardiovascular risk factors in adults.*

• *Children with primary dyslipidemia may develop early cardiac dysfunction.*

**What is New:**

• *This study demonstrates a signifi cant association between epicardial adipose tissue thickness and left ventricular function in children.*

• *It highlights the potential role of epicardial fat as an early marker of cardiac involvement in pediatric dyslipidemia.*

## Introduction

Dyslipidemias can result from primary lipoprotein metabolism changes due to different genetic causes (primary dyslipidemias) or as a consequence of exogenous factors or other pathologies (secondary dyslipidemias) [1]. Primary dyslipidemia can be due to familial disorders as well. Autosomal dominant mutations cause most cases of familial hypercholesterolemia in LDL receptors, which causes an elevation in LDL-C levels. Other mutations in the cholesterol pathway have been identified but are less common [2].

Visceral adiposity, defined as fat deposition around internal organs, is metabolically active and represents an important risk factor for developing metabolic syndrome [3–5].

The heart and vessels are surrounded by layers of adipose tissue, which is a complex organ composed of adipocytes, stromal cells, macrophages, and a neuronal network, all nourished by a rich microcirculation. These layers of adipose tissue surrounding the heart can be subdivided into intra- and extra-pericardial fat. Their thicknesses and volumes can be quantified by echocardiography, CT, or magnetic resonance imaging [6–8].

Epicardial fat is located between the outer wall of the myocardium and the visceral layer of pericardium. Epicardial adipose tissue (EAT) is a component of visceral adiposity and mediates cardiac function and atherosclerosis via expression of several bioactive molecules [9, 10].

Studies in adults suggest that increased EAT results in greater cytokine production in the fat directly surrounding coronary vessels, including tumor necrosis factor-α and several interleukins. These cytokines contribute to atherosclerosis by increasing lipolysis, inflammation, and endothelial dysfunction. Endothelial dysfunction coupled with migration of macrophages and other immune cells further exacerbates plaque formation and may eventually cause disruption of the intima and flow within the lumen [6, 8]*.*

## Aim of the study

To evaluate the significance and relationship between epicardial fat thickness (EFT) and familial dyslipidemia and to detect and classify common types of familial dyslipidemia.

## Methodology

This study is a prospective case control study was conducted at Assiut University Children’s Hospital, Assiut, Egypt, between Sep 2023 to Aug 2025. In this study, we investigated 21 patients-aged more than 1 month and till age of 18 years with a diagnosis of familial dyslipidemia. Another 21 apparently healthy volunteers were enrolled in the study as the control group. However, patients who had poor echo window: chest deformities, chronic lung disease, pericardial and/or pleural effusion on transthoracic echocardiography, chronic kidney disease congenital heart diseases, Diabetes that cause 2ry dyslipidemia and liver diseases that cause 2ry dyslipidemia were excluded in the study. The control group included asymptomatic healthy children matched by age, sex, and body mass index (BMI) from the pediatric cardiology outpatient clinic of our Pediatric Department, selected from children who were sent for functional heart murmurs and whose echocardiography was normal.

Sample size: Based on determining the main outcome variable, the estimated minimum required sample size is 42 patients (21 patient in each group) The sample was calculated using G*power software 3.1.9.2., based on the following assumptions: Main outcome variable is the significance of the relationship between epicardial fat thickness in cases with familial dyslipidemia and healthy controls. Based on clinical experience we expected to find large effect size difference between 2 groups Main statistical test is independent t-test to detect the difference between the 2 groups. Alpha = 0.05 Power = 0.80 Effect size = 0.8.

All children enrolled in the study were subjected to a full history taking for demographic as Age—Sex (female/male), Social history would include tobacco use or specific details about diet & Family history. clinical examination with special emphasis on Xanthomas is deposits of lipids on the skin and sometimes subcutaneous tissue, weight, height, BMI (kg/m2), Waist circumference (cm), systolic blood pressure (SBP), and diastolic blood pressure (DBP).

Total cholesterol (TC), Low-density lipoprotein cholesterol (LDL-C), High- density lipoprotein cholesterol (HDL-C), and Serum Triglyceride (TG) were recorded for all children enrolled in the study [11]. Dyslipidemia was considered present if one or more of lipid or lipoprotein levels are abnormal.

Transthoracic echocardiographic examination with a simultaneous ECG tracing was performed for all children included in the study in both supine and left lateral position using a commercially available device Philips Envisor ultrasound system (Philips Medical Systems, Andover, MA, USA; monitor module manufactured in Saronno, Italy; Model No. MCMD02AA, Type No. M2540-66500) equipped with 21350 A PHILIPS HP S8 sector array transducer (2–8 MHz), (Andover, MA, USA Serial number: US503086425) according to the American Society of Echocardiography recommendations. Two experienced pediatric echocardiographers blinded to the children’s clinical picture measured the EFT and the echocardiographic parameters**.**

Conventional 2D-guided M-mode echocardiography was done for the measurement of the cardiac dimensions (wall thickness and chamber size) and left ventricular (LV) circumferential systolic function [ejection fraction (EF) and fractional shortening (FS)]. Conventional pulsed-wave Doppler echocardiography was used for evaluation of LV diastolic function through mitral inflow velocities obtained in the apical four-chamber view [peak early diastolic filling velocity (*E*), peak late diastolic filling velocity (*A*), the *E*/*A* ratio, and deceleration time (DT) of early mitral flow] [12].

Echocardiographic measurement of EFT was performed for all subjects included in the study according to the guidelines of American Society of Echocardiography [8]. Standard parasternal long-axis and short-axis views from 2D images, with optimal cursor beam orientation in each view, were obtained for the most accurate measurement of EFT on the right ventricle. EFT was measured perpendicularly on the free wall of the right ventricle at end systole, using the aortic annulus as an anatomic landmark. For midventricular parasternal short-axis assessment, maximum EFT was measured on the right ventricular free wall along the midline of the ultrasound beam perpendicular to the interventricular septum at midchordal and tip of the papillary muscle level, as anatomic landmarks. The average value of 3 cardiac cycles from each echocardiographic view was determined.

### Statistical analysis

Data were analyzed using SPSS version 26 (IBM Corp., Armonk, NY, USA). Qualitative data were presented as numbers and percentages, while quantitative data were expressed as mean ± standard deviation. Statistical tests included the Chi-square test for categorical variables, independent-sample t-test for continuous variables, and Pearson’s correlation to assess relationships between epicardial fat thickness (EFT) and lipid or echocardiographic parameters. Multivariable linear regression was performed to identify independent predictors of EFT after adjusting for age, sex, BMI, and HDL-C. A *p*-value < 0.05 was considered statistically significant.

### Ethical considerations

This study was conducted in accordance with the ethical standards of the Assiut Medical Ethical Review Board (IRB No. 04–2023–200448) and in accordance with the Declaration of Helsinki. All patients included in this study received their medical care free of charge. Refusal to participate in the study did not affect the quality of medical care provided. Written informed consent was obtained from the parents or legal guardians of all participants, and confidentiality of the collected data was ensured.

An official written administrative approval was obtained from the Dean of the Faculty of Medicine, Assiut University Hospital. The title and objectives of the study were explained to ensure full cooperation.

## Results

A total of 42 participants were included in the study, divided equally into two groups**:** 21 patients with dyslipidemia and 21 age- and sex-matched healthy controls**.** All participants underwent thorough clinical evaluation, including laboratory assessment of lipid profile parameters (cholesterol, triglycerides, HDL, and LDL) and echocardiographic examination, with a specific focus on measuring epicardial fat thickness (EFT) in both the parasternal long axis (PSLAX) and parasternal short axis (PSSAX) views**.**

The case group demonstrated significantly higher epicardial fat thickness (EFT) measured by echocardiography (*p* = 0.018), larger left atrial diameter (LA) (*p* = 0.031), and larger ascending aortic diameter (AAO) (p = 0.013) compared to the control group. No statistically significant differences were observed between the two groups regarding mitral E velocity, mitral E/A ratio, pulmonary artery diameter, interventricular septum thickness, left ventricular end-systolic diameter, left ventricular posterior wall thickness, or fractional shortening (*p* > 0.05 for all). These findings indicate selective structural cardiac changes in the case group as shown in Table 1,

**Table 1:** Echocardiographic data among the studied groups for each group

Variables	parameters	Case Group (*N *= 21)	Control Group (*N* = 21)	*P*-value
EFT BY ECHO (mm)	Mean ± SD	2.879 ± 0.935	2.288 ± 0.574	0.018*
	Min–max	1.750–4.450	1.100–2.900	
MITRAL E (cm/s)	Mean ± SD	120.571 ± 38.373	113.571 ± 31.494	0.687
	Min–max	73–190	73–198	
MITRAL A (cm/s)	Mean ± SD	70.71 ± 18.87	65.95 ± 16.62	0.402
	Min–max	73–103	40–101	
MITRAL E/A	Mean ± SD	1.7 ± 0.391	1.756 ± 0.359	0.860
	Min–max	1.02—2.7	1.3–2.6	
PA (mm)	Mean ± SD	14.986 ± 2.767	14.424 ± 2.829	0.434
	Min–max	11.3—19.1	11.3–20.2	
LA (mm)	Mean ± SD	21.452 ± 3.862	18.029 ± 5.226	0.031*
	Min–max	16.8–28	10.2–25	
AO (mm)	Mean ± SD	17.538 ± 3.123	14.338 ± 3.322	0.013*
	Min–max	11.7—21.6	5.5–19.5	
RV (mm)	Mean ± SD	8.98 ± 2.97	9.21 ± 3.11	0.8
	Min–max	3.04- 14.92	3.00—15.42	
IVSD (mm)	Mean ± SD	6.381 ± 2.847	6.867 ± 6.952	0.130
	Min–max	2–13	2–27.500	
LVEDD (mm)	Mean ± SD	29.010 ± 7.411	34.995 ± 14.648	0.279
	Min–max	18.7–38	17–72	
LVESD (mm)	Mean ± SD	20.457 ± 3.252	16.362 ± 7.754	0.070
	Min–max	11.7–24.6	2.5–26.6	
LVPWD (mm)	Mean ± SD	5.167 ± 1.346	4.471 ± 1.216	0.226
	Min–max	3.1–7.7	2.3–7.1	
LVFS (%)	Mean ± SD	41.5 ± 5.310	39.810 ± 4.397	0.433
	Min–max	32–51	33–46	

The echocardiographic parameters in the case group categorized according to z-scores, which were used to adjust measurements for age-related variations. The normal reference range was defined as z-scores between − 2 and + 2, while values below − 2 and above + 2 were considered below normal and above normal, respectively as shown in Table 2.

**Table 2:** Echocardiographic data in case group according to z score

Variables	Below z score	Normal	Above z score
PA (mm), n%	4 (19.05%)	17 (80.95%)	0 (0%)
AAO (mm)	0 (0%)	19 (90.48%)	2 (9.52%)
IVSD (mm)	4 (19.05%)	13 (61.91%)	4 (19.05%)
LVEDD (mm)	10 (47.62%)	11 (52.38%)	0 (0%)
LVESD (mm)	5 (23.81%)	15 (71.43%)	1 (4.76%)
LVPWD (mm)	0 (0%)	20 (95.24%)	1 (4.76%)

The case group showed significantly higher levels of cholesterol**,** triglycerides (TG)**,** and low-density lipoprotein (LDL) compared to the control group (*p *< 0.001 for all), indicating a markedly altered lipid profile. Although high-density lipoprotein (HDL) was lower in the case group, the difference did not reach statistical significance (*p *= 0.059). These findings suggest that dyslipidemia is more prominent among the case group, which may have clinical implications for cardiovascular risk as shown in Table 3.

**Table 3:** Lipid profile data among the studied groups for each group

Variables	Parameters	case Group (*N* = 21)	Control Group (*N* = 21)	*P*-value
Total cholesterol (mg/dL)	Mean ± SD	332.952 ± 222.320	145.390 ± 16.975	< 0.001*
	Min–max	139–832	132.800–216	
Triglycerides (mg/dL)	Mean ± SD	391.381 ± 251.558	114.181 ± 22.238	< 0.001*
	Min–max	100–844	76.9–149.6	
High-Density Lipoprotein cholesterol (mg/dL)	Mean ± SD	44.467 ± 27.690	50.786 ± 6.503	0.059
	Min–max	20–123	42.6–63.2	
Low-Density Lipoprotein cholesterol (mg/dL)	Mean ± SD	154.347 ± 130.361	68.129 ± 13.198	< 0.001*
	Min–max	75.8–634	50.3–90.8	

**Fig. 1:**
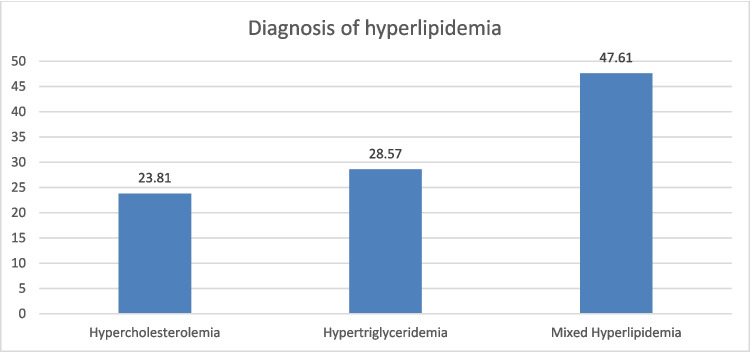
Diagnosis of hyperlipidemia among the case group

Among the case group (*n* = 21), mixed hyperlipidemia was the most prevalent diagnosis, accounting for 47.61% of cases, followed by hypertriglyceridemia at 28.57% and hypercholesterolemia at 23.81% as shown in Fig. 1.

**Fig. 2:**
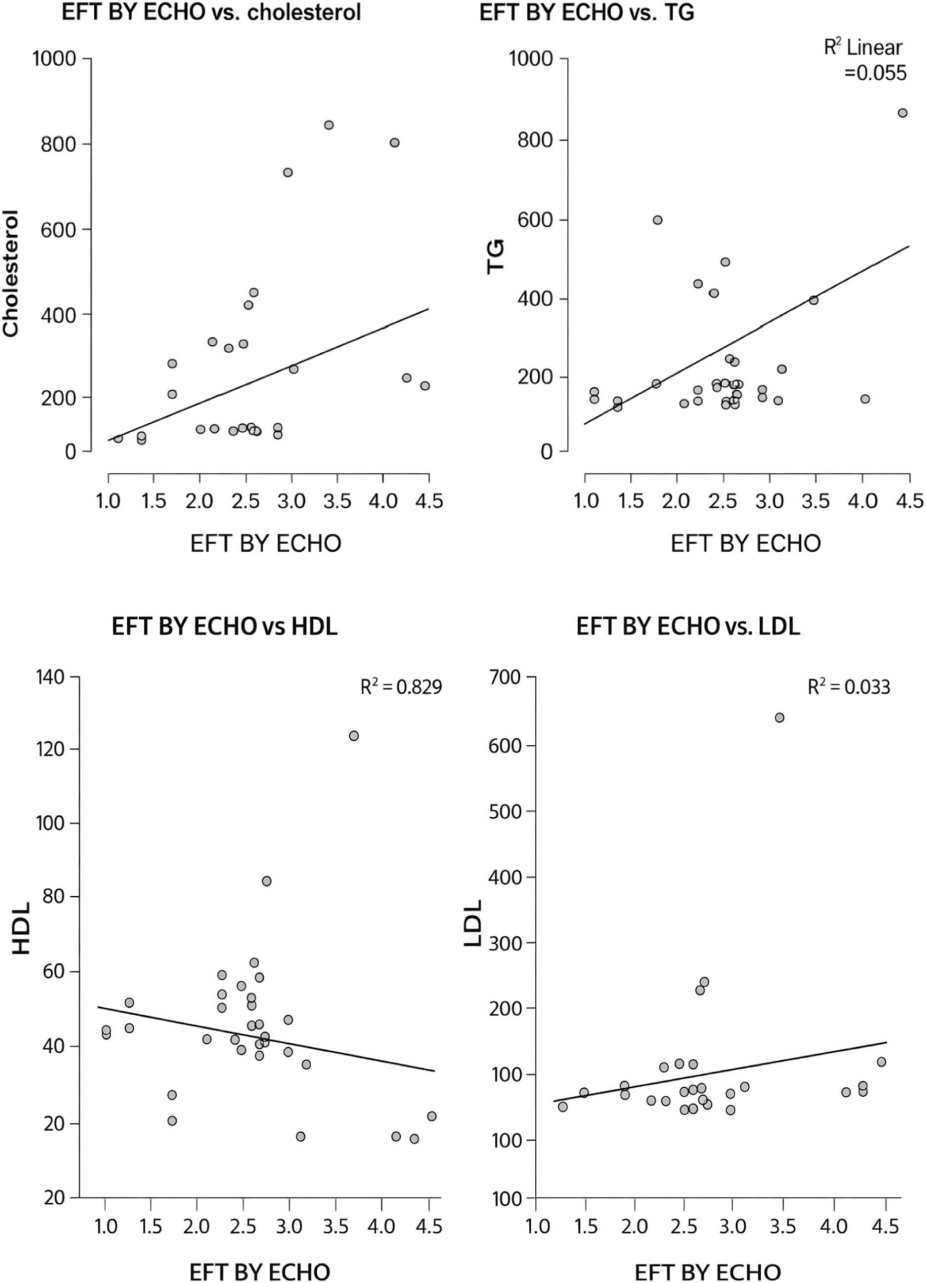
Correlation between EFT By ECHO and lipid profile

No significant correlations were found between epicardial fat thickness (EFT) and any component of the lipid profile among the case group. However, a weak negative correlation was observed between EFT and HDL levels, which approached significance and may suggest a potential inverse relationship worth exploring in larger studies. Correlations with total cholesterol, triglycerides, and LDL were weak and not statistically significant as shown in Table 4 and Fig. 2.

**Table 4:** Correlation between EFT measured by ECHO and lipid profile among the case group

Lipid profile	R -value	*P*-value
Total cholesterol (mg/dL)	0.243	0.121
Triglycerides (mg/dL)	0.235	0.134
High-Density Lipoprotein cholesterol (mg/dL)	−0.288	0.064
Low-Density Lipoprotein cholesterol (mg/dL)	0.183	0.258

*TG* triglycerides, *TC* total cholesterol, *LDL* low-density lipoprotein cholesterol, *HDL*

high-density lipoprotein cholesterol,* EFT* epicardial fat thickness

**Fig. 3:**
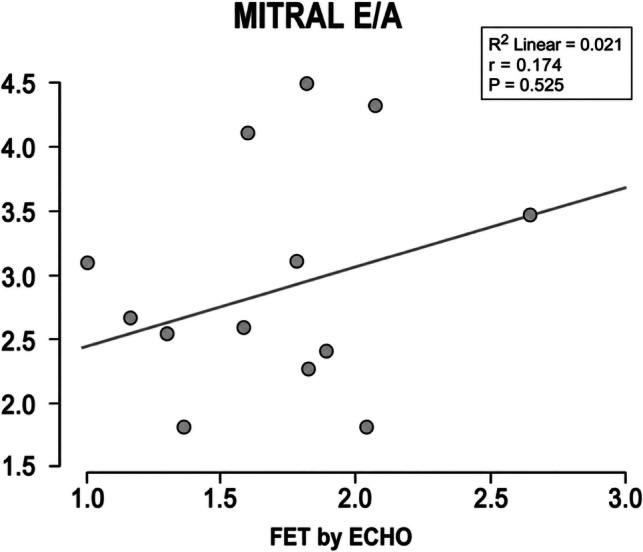
Correlation between EFT by ECHO and Mitral E\A. FS Fractional Shortening, EFT epicardial fat thickness

**Fig. 4:**
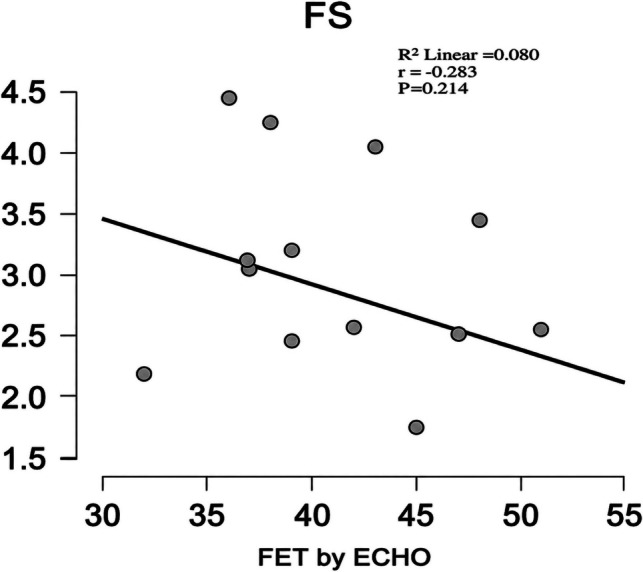
Correlation between EFT by ECHO and FS

Epicardial fat thickness (EFT) measured by echocardiography showed no significant correlations were observed between EFT and fractional shortening (FS) (r = –0.283, *p* = 0.214) or mitral E/A ratio (r = 0.147, *p* = 0.525) as shown in Table 5 and Figs. 3 and 4.

**Table 5:** Correlation between EFT by ECHO and FS and Mitral E\A among the case group

	R -value	*P*-value
FS	−0.283	0.214
MITRAL E/A	0.147	0.525

*E *mitral peak early diastolic filling velocity, *A *mitral peak late diastolic filling velocity, *EFT *epicardial fat thickness

In the multivariable linear regression analysis assessing the relationship between lipid profile parameters and epicardial fat thickness (EFT) adjusted for age, sex and BMI.

The level of significance in this study was set to *P* value less than 0.05 Among the case group, triglyceride (TG) level was the only significant predictor of EFT (B = 0.002, *p *= 0.028), indicating that higher TG levels are independently associated with increased EFT. Cholesterol, HDL, and LDL levels did not show statistically significant associations with EFT (*p* > 0.05). Among all predictors, TG also had the highest standardized beta coefficient (β = 0.437), reflecting its relatively stronger contribution to the model as shown in Table 6.

**Table 6:** Multivariable linear regression of lipid profile in Relation to EFT by ECHO among the case group

Variable	B (Unstandardized)	Beta (Standardized)	T	P	95% CI
(Intercept)	2.164	-	3.681	< 0.001*	0.97 −3.35
Total cholesterol	0.001	0.253	1.102	0.276	- 0.008 −0.003
Triglycerides (TG)	0.002	0.437	2.288	0.028*	0.0001 −0.003
High-Density Lipoprotein cholesterol	- 0.005	- 0.127	- 0.396	0.695	- 0.032 −0.022
Low-Density Lipoprotein cholesterol	< 0.001	0.065	0.165	0.870	- 0.006 −0.007

## Discussion:

A total of 42 participants were included in the study, divided equally into two groups: 21 patients with dyslipidemia and 21 age, sex and BMI-matched healthy controls. All participants underwent thorough clinical evaluation, including laboratory assessment of lipid profile parameters (cholesterol, triglycerides, HDL, and LDL) and echocardiographic examination, with a specific focus on measuring epicardial fat thickness (EFT) in both the parasternal long axis (PSLAX) and parasternal short axis (PSSAX) views.

In this single-center case–control study of children more than 1 month and till age of 18 years that attended Assiut University Children Hospital, no significant differences were found between cases and controls in terms of age, sex, BMI, hemodynamic or anthropometric parameters. This baseline equivalence strengthened the validity of EFT comparisons and reduces confounders. Recent pediatric reviews confirmed EFT can differ independently of BMI, making matched groups essential [13].

Regarding Epicardial Fat Thickness (EFT) and Cardiac Structures, EFT was significantly higher in dyslipidemic children (mean 2.879 mm) vs. controls (2.288 mm), *p *= 0.018. Additionally, LA and aortic root dimensions were larger in cases, while systolic and diastolic functions remained similar. Multiple pediatric studies reported elevated EAT/EFT in children with cardiometabolic risk factors, including obesity and type 1 diabetes, corroborating our findings. Moreover, EFT measured by echocardiography (PSLAX/PSSAX) remained a validated, non-invasive proxy for cardiac visceral fat. Structural changes preceding functional impairment echo reports of early remodeling with preserved ventricular performance [14–16].

In the present study, with familial dyslipidemia, epicardial fat thickness (EFT) measured by echocardiography was significantly higher in dyslipidemic patients than in age- and sex-matched controls, despite comparable anthropometrics and hemodynamics. This primary finding was consistent with recent pediatric literature identifying epicardial adipose tissue (EAT) as an early, echocardiographically accessible marker of cardiometabolic risk in youth, across a spectrum of metabolic states (obesity, diabetes, and dyslipidemia). Multiple contemporary reviews and cohorts showed that increased EAT/EFT in children associates with adverse risk phenotypes and subclinical cardiac adaptation, supporting its use as a non-invasive risk stratifier [17].

Dyslipidemic children had significantly higher total cholesterol, triglycerides, and LDL-C (p < 0.001), though HDL was non-significantly lower (*p *= 0.059). These findings were in strong agreement with Dönmez and Bulut reported that EFT was significantly higher in FH patients compared to controls (6.30 ± 2.31 mm vs. 4.94 ± 0.94 mm, p < 0.001), and identified LDL-C as the only independent predictor of EFT in multivariate analysis. This parallels our results, highlighting LDL-C as the most influential lipid fraction associated with EFT increase [18].

Mixed hyperlipidemia was most prevalent (47.6%), followed by hypertriglyceridemia (28.6%) and hypercholesterolemia (23.8%).

The dominance of mixed dyslipidemia mirrors recent pediatric trends, which indicate combined lipid abnormalities are increasingly common and linked with ectopic fat deposition (inc. EAT) [15, 19].

In the present study, no significant correlations were observed between epicardial fat thickness (EFT) and total cholesterol (TC), triglycerides (TG), or low-density lipoprotein cholesterol (LDL-C). However, a borderline inverse correlation was noted with high-density lipoprotein cholesterol (HDL-C) (r = − 0.288, *p *= 0.064). This finding suggested that EFT may not consistently reflect traditional lipid fractions but could be more closely associated with the protective role of HDL against visceral fat accumulation. These results diverged from observations in familial hypercholesterolemia (FH) populations, where EFT has shown strong positive correlations with LDL-C, likely reflecting the pronounced atherogenic lipid burden characteristic of FH patients [20].

The borderline inverse trend with HDL observed in our cohort is consistent with evidence that reduced HDL levels are associated with greater visceral adiposity and impaired cardiometabolic protection.

Similarly, Ferrara et al., highlighted that reduced HDL impairs its protective anti-inflammatory and antioxidant functions, thereby facilitating ectopic fat accumulation and increasing cardiovascular risk. They also noted that dyslipidemia, particularly the combination of low HDL and elevated triglycerides, is strongly associated with dysfunctional adipose tissue signaling. This supports the interpretation that HDL may play a central regulatory role in the relationship between ectopic fat depots, dyslipidemia, and cardiovascular disease [21].

In the current study, there was no significant correlation between EFT and functional indices (FS, E/A). Abd ElBaky et al., reported children with Type 1 diabetes had increased EFT associated with early predictors of dysfunction; yet conventional FS and E/A were often similar to controls, suggesting subclinical changes not detected by basic indices. And this reinforced that metabolic disease in children can elevate EFT and produce structural signs before FS/E-A abnormalities [22].

## Conclusion

The study found that children with dyslipidemia had significantly higher total cholesterol, triglycerides, and LDL levels than matched healthy controls, with mixed hyperlipidemia being the most common pattern. Echocardiography showed increased epicardial fat thickness (EFT) and larger left atrial and aortic diameters, indicating early cardiac remodeling. Multivariable analysis identified serum triglycerides as the only independent predictor of EFT. Although no significant correlations were found with other lipid parameters, a negative trend with HDL was noted. Overall, EFT appears to be a sensitive marker for early cardiovascular risk in dyslipidemic children, primarily influenced by triglyceride levels.

## Limitations of the study

- Small sample size: Only 21 patients per group, limiting statistical power and increasing risk of overfitting in multivariable analyses. Findings, including triglycerides as an independent predictor of EFT, should be interpreted cautiously.
- Cross-sectional design: No longitudinal follow-up to assess long-term cardiovascular outcomes.
- Broad age range: Participants ranged from infancy to 18 years. Cardiac dimensions vary with age, and indexing to body surface area (BSA) or using Z-scores would strengthen interpretation.
- Limited functional assessment: Only conventional echocardiographic indices (fractional shortening and mitral E/A ratio) were used. These are load-dependent and may not detect early myocardial dysfunction. Speckle-tracking echocardiography and GLS would provide more sensitive assessment, but the software was not available in our device

## Data Availability

No datasets were generated or analysed during the current study.
